# Trop2-Based Antibody–Drug Conjugates: Emerging Strategy and Progress in Triple-Negative Breast Cancer Therapy

**DOI:** 10.3390/curroncol33020092

**Published:** 2026-02-03

**Authors:** Tong Li, Tao Zhang, Yongxia Dang, Yilin Lin, Xiaotong Li, Xiaoling Ling

**Affiliations:** 1 The First Clinical Medical College, Lanzhou University, No. 199 Donggang West Road, Lanzhou 730000, China; lit2024@lzu.edu.cn (T.L.); dangyx2023@lzu.edu.cn (Y.D.); linyl2024@lzu.edu.cn (Y.L.); lxiaotong2024@lzu.edu.cn (X.L.); 2 Department of Oncology, The First Hospital of Lanzhou University, No. 1 Donggang West Road, Lanzhou 730000, China; zhangt18@lzu.edu.cn

**Keywords:** triple-negative breast cancer (TNBC), Trophoblast cell-surface antigen 2 (Trop2), antibody–drug conjugates (ADCs), targeted therapy, clinical research

## Abstract

Triple-negative breast cancer (TNBC) accounts for 15–20% of invasive breast cancers and represents a highly heterogeneous, aggressive subtype with poor prognosis and limited treatment options, necessitating the identification of novel therapeutic targets to improve clinical outcomes. Trophoblast cell-surface antigen 2 (Trop2), a calcium signal transducer, is frequently overexpressed in TNBC (approximately 88% of cases) while exhibiting minimal expression in normal tissues. Its overexpression is significantly associated with tumor invasion, metastasis, and unfavorable prognosis. Antibody–drug conjugates (ADCs) targeting Trop2 have demonstrated significant clinical potential. This article systematically reviews the clinical research progress of Trop2-targeted ADCs, aiming to provide evidence-based insights for improving the prognosis of TNBC patients.

## Simple Summary

With the aim of providing evidence-based insights for improved prognosis in TNBC patients, this article conducts a systematic review of the clinical research progress of Trop2-targeted ADCs. Trop2-targeted therapies are set to become a cornerstone of precision medicine for solid tumors, thereby paving the way for superior treatment options for patients.

## 1. Introduction

As the most common malignant tumor in women, breast cancer constitutes 32% of new cancer cases according to the 2025 Global Cancer Statistics Report, representing the leading component of female cancer incidence [1]. Among breast cancer subtypes, triple-negative breast cancer (TNBC) is characterized by the lack of established therapeutic targets and insensitivity to endocrine and targeted therapies. This results in limited treatment options and a typically poor prognosis [2,3,4]. It is important to consider immunotherapy and Poly (ADP-ribose) Polymerase inhibitors (PARPi) as standard treatment options, which have been shown to improve the prognosis of TNBC. However, a substantial unmet clinical need remains for the broader TNBC population, particularly for patients with treatment-resistant or recurrent/metastatic disease [5]. This pressing situation underscores the urgent necessity to explore novel therapeutic targets and mechanisms of action.

Antibody–drug conjugates (ADCs), which combine a targeted antibody with a potent cytotoxic payload, offer a paradigm shift in cancer therapy by enabling precise delivery of chemotherapy to tumor cells [6]. Against this backdrop, Trophoblast cell-surface antigen 2 (Trop2), a type I transmembrane glycoprotein belonging to the tumor-associated calcium signal transducer (TACSTD) family, has emerged as an ideal target for ADC development [7,8]. Its suitability as a target rests on several key features: First, Trop2 expression is extremely low in normal tissues but is stably and highly expressed on the membrane of various epithelial-derived malignancies, including TNBC, where its positivity rate reaches approximately 88%. This differential expression provides a favorable therapeutic window. Second, its overexpression correlates with aggressive tumor behavior, metastatic potential, and poor clinical prognosis [6,7,8]. Mechanistically, Trop2 promotes tumor cell proliferation, migration, and invasion by regulating key signaling pathways such as the Phosphatidylinositol 3-Kinase (PI3K)/Protein Kinase B (AKT) and Mitogen-activated Protein Kinase (MAPK) [9,10]. Furthermore, the Trop2-antibody complex undergoes efficient internalization, ensuring the precise delivery and intracellular release of the conjugated cytotoxic payload, thereby completing the “target binding-internalization-killing” cycle that underlies ADC efficacy [11,12]. These intrinsic biological properties collectively form the molecular foundation for the remarkable therapeutic activity of Trop2-targeted ADCs.

To date, Sacituzumab govitecan (SG), as the first Trop2-targeted ADC approved for metastatic TNBC (mTNBC) treatment, has demonstrated significant survival benefits in clinical studies [13]. Additionally, next-generation agents such as datopotamab deruxtecan (Dato-DXd) and sacituzumab tirumotecan (SKB-264) also show promise [14,15]. Notably, differences in their design—including linker chemistry, payload class, and drug-to-antibody ratio (DAR)—directly translate into distinct efficacy and safety profiles, as well as potential resistance mechanisms. Therefore, systematically elucidating their mechanisms of action, critically synthesizing and comparatively analyzing of their clinical data, and a thorough discussion of current challenges and future directions are essential for advancing precision therapy in TNBC. This review aims to provide a comprehensive overview of the current landscape and progress in Trop2-targeted ADCs development for TNBC, with the goal of offering an evidence-based reference to inform both clinical practice and future research.

## 2. Molecular Structure and Biological Characteristics of Trop-2

**Figure 1:**
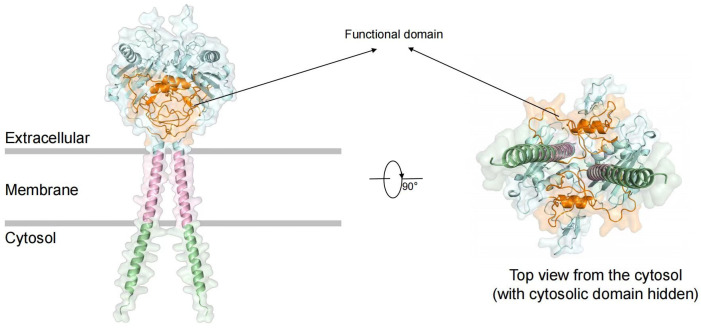
Schematic structure of Trop2 protein (viewed from the cytosolic side with the intracellular domain hidden). In the figure, the Extracellular domain is colored blue; the Membrane region is colored pink; and the Cytoplasmic domain is colored green. the functional domain shown is Thyroglobulin type-1, colored orange. Trop2 is a type I transmembrane glycoprotein comprising an extracellular domain (ECD), a single-pass transmembrane α-helix (TM), and an intracellular domain (ICD). The ECD contains thyroglobulin-like domains and N-glycosylation sites, while the ICD harbors a PIP2-binding motif. Its homomeric oligomerization potentiates signal transduction. The figure was created using the PyMOL Molecular Graphics System, Version 3.0.3.

Trop2 is a type I transmembrane glycoprotein encoded by the *TACSTD2* gene located on chromosome 1p32.1, and it consists of 323 amino acid residues [16]. The protein comprises three critical structural domains: an extracellular domain (ECD) containing thyroglobulin-like domains and cysteine-rich regions with four N-glycosylation sites; a single-pass transmembrane α-helix (TM); and an intracellular domain (ICD) harboring a conserved PIP2-binding motif (GAPALPPK) that participates in the regulation of signal transduction (Figure 1) [17,18]. Functionally, Trop2 mediates calcium ion signaling to activate both the PI3K/AKT and MAPK pathways, with its unique oligomerization properties significantly enhancing the intensity of signal transduction [19]. Preclinical studies have shown that Trop2-targeted Fab fragments effectively inhibit the growth of TNBC xenograft tumors, exhibiting potent antitumor activity [20]. Clinical investigations reveal that Trop2 expression positivity reaches 88% [Trop2 Immunohistochemistry (IHC) 2+/3+] in patients with mTNBC, and Trop2 serves as an independent prognostic factor significantly associated with poor clinical outcomes (hazard ratio [HR] = 1.010, 95% confidence interval [CI]: 1.001–1.020, *p* < 0.05) [21]. In contrast, Trop2 is expressed at low levels or shows limited expression in normal tissues (such as normal breast epithelium, intestinal mucosa, and pancreatic ductal epithelium), where it is present only in small amounts within certain differentiated epithelial cells. Consequently, Trop2 has emerged as a promising therapeutic target in TNBC and various other malignancies, with multiple Trop2-targeting ADC candidates currently under clinical investigation.

## 3. Molecular Mechanisms of Trop2-Targeted ADCs

### 3.1. Basic Components and Intracellular Delivery

**Figure 2:**
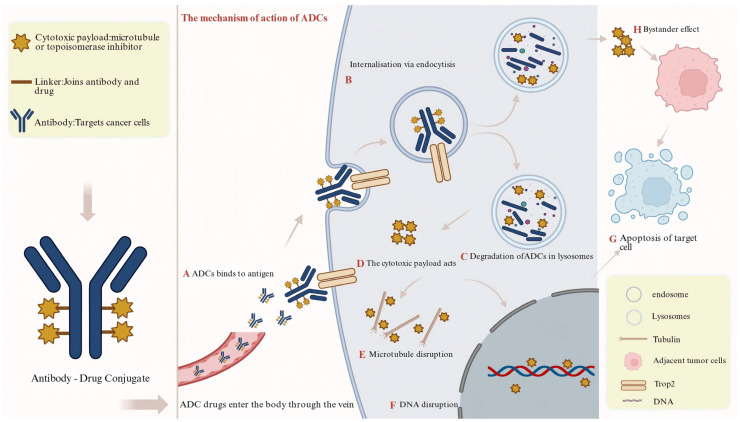
Mechanism of action of Trop2-targeted ADCs. (1) Targeted Binding: The antibody component of the ADC specifically recognizes and binds to antigens on the surface of tumor cells. (2) Internalization & Trafficking: The resulting antigen–antibody complex is internalized into the cell via endocytosis. (3) Payload Release: Within the lysosome, the linker is cleaved, releasing the highly potent cytotoxic payload. (4) Effector Function: The released drug (e.g., a microtubule disruptor or a DNA-damaging agent) acts on its intracellular target, disrupting essential cellular functions and ultimately inducing apoptosis in the target cell. Furthermore, certain ADCs can exert a “bystander effect (BE)”, where the released drug diffuses to and kills adjacent tumor cells, enhancing the overall antitumor activity. Figure created with BioRender.com.

Trop2-targeted ADCs comprise three critical components: a monoclonal antibody that specifically recognizes the ECD of Trop2, a cleavable linker (e.g., the pH-sensitive CL2A or the protease-sensitive Val-Cit sequence), and a highly potent cytotoxic payload (e.g., the topoisomerase I (Top I). inhibitor SN-38 or the tubulin inhibitor DM4) [22,23]. These therapeutic agents enter tumor cells through receptor-mediated endocytosis and follow the endosomal–lysosomal trafficking route. Within the acidic lysosomal compartment, proteases such as cathepsin B cleave the linker, releasing the active toxin. The freed payload then induces DNA damage or disrupts microtubule function, ultimately triggering tumor cell death [17,18,19] (Figure 2).

### 3.2. Direct Cytotoxic Mechanisms

The released payloads directly induce tumor cell death by interfering with core cellular processes. Based on their molecular targets, the primary mechanisms are categorized as follows.

#### 3.2.1. Mechanism of Topoisomerase I Inhibitors

Top I Inhibitors (e.g., SN-38, DXd, T030): These agents exert cytotoxicity by stabilizing the topoisomerase I-DNA cleavage complex (Top1cc), leading to replication fork collapse, DNA double-strand breaks, and activation of apoptotic pathways [24]. Although sharing this core mechanism, different derivatives (e.g., SN-38, DXd, T030) exhibit distinct pharmacological properties such as membrane permeability and metabolic stability, which directly influence the potency of their bystander effect and clinical toxicity profiles [13,17,25,26] (Table 1).

#### 3.2.2. Mechanism of Tubulin Inhibitors

Tubulin inhibitors, represented by DM4, bind with high affinity to β-tubulin subunits, disrupting microtubule dynamics and causing mitotic spindle defects. This aberrant spindle assembly activates the spindle assembly checkpoint (SAC), arresting the cell cycle at the G2/M phase and eventually inducing cell death [20].

### 3.3. Indirect Antitumor Effects

#### 3.3.1. Bystander Effect

The Bystander Effect (BE) is crucial for ADCs to overcome tumor antigen heterogeneity. Hydrophobic payloads (e.g., SN-38) released inside target cells can diffuse across the plasma membrane and kill neighboring tumor cells that express low or no Trop2. This mechanism significantly expands the therapeutic reach of ADCs in antigenically heterogeneous tumors [21].

#### 3.3.2. Immunomodulation and Remodeling of the Tumor Microenvironment

ADCs induced tumor cell death can exhibit immunogenic features, resulting in the release of damage-associated molecular patterns (DAMPs) such as High mobility group box-1 protein (HMGB1) and ATP. These signals promote the maturation and activation of APCs (e.g., dendritic cells, marked by upregulation of CD86) and enhance cytokine production (e.g., interferon-gamma [IFN-γ]) by effector CD8+ T cells, thereby stimulating an adaptive anti-tumor immune response [24]. Moreover, by effectively clearing tumor cells, ADCs indirectly suppress cancer-cell-driven pro-survival and immunosuppressive pathways (e.g., NF-κB, Wnt/β-catenin) in the tumor microenvironment (TME) while reducing the secretion of immunosuppressive factors such as interleukin-6 (IL-6) and Vascular Endothelial Growth Factor (VEGF). This remodeling creates a more favorable context for combination therapies, including immune checkpoint inhibitors [27].

### 3.4. Summary: A Synergistic Multi-Effect Framework

In summary, Trop2-targeted ADCs achieve precise drug delivery and exert a coordinated, multi-layered impact encompassing direct cytotoxicity, the BE, and immunomodulation of the TME. Together, these actions establish an integrated therapeutic framework characterized by precision targeting, broad coverage, and sustained control. This provides the molecular foundation for the profound and durable clinical responses observed in breast cancer treatment. The following sections will explore how these mechanistic principles are translated into the clinical reality of three leading Trop2-targeted ADCs and how their distinct molecular architectures correlate with observed differences in efficacy and safety profiles in TNBC trials.

## 4. Research Progress of Trop2-ADCs in TNBC Treatment

### 4.1. Sacituzumab Govitecan (SG): From Mechanistic Innovation to Clinical Validation and Expansion

#### 4.1.1. Design Rationale and Mechanistic Foundation

Sacituzumab govitecan (SG) (Table 2), the first globally approved Trop2-targeted ADC, consists of a humanized IgG1 monoclonal antibody conjugated to SN-38 (the active metabolite of irinotecan) via a hydrolyzable CL2A linker, with a DAR of 7.6 [24]. Its design underpins a dual mechanism of action: intracellular SN-38 release following lysosomal degradation, and extracellular release via cleavage of the pH-sensitive CL2A linker in the acidic TME [27]. This strategy facilitates potent cytotoxic activity and a pronounced bystander effect, contributing to its clinical efficacy in pretreated mTNBC [28].

#### 4.1.2. Pivotal Clinical Efficacy and Safety Data

In the treatment of mTNBC, SG has demonstrated substantial antitumor efficacy with a manageable safety profile. Pivotal data from the Phase I/II IMMU-132-01 trial (NCT01631552) [25] and Phase III ASCENT study (NCT02574455) [29] demonstrated that in patients with mTNBC, SG yield an Objective Response Rate (ORR) of 33%,with a median Progression-free survival (mPFS) of 5.5 months and a median Overall Survival (mOS) of 13.0 months. Compared with treatment of physician’s choice (TPC) chemotherapy (TPC: eribulin, vinorelbine, gemcitabine, or capecitabine), SG significantly improves both mPFS (5.6 months vs. 1.7 months; HR = 0.39, 95% CI: 0.30–0.49) and mOS (12.1 months vs. 6.7 months; HR = 0.48, 95% CI: 0.38–0.59). The U.S. Food and Drug Administration (FDA) and China’s National Medical Products Administration (NMPA) have approved SG for the second-line or later treatment of mTNBC [30,31]. The Phase III ASCENT-03 study (NCT05382299) evaluated SG versus TPC (Paclitaxel, nab-Paclitaxel or Gemcitabine + Carboplatin) as first-line treatment for mTNBC [32]. Results showed SG significantly prolonged mPFS (9.7 vs. 6.9 months; HR = 0.62) and median duration of response (mDOR) (12.2 vs. 7.2 months), although ORRs were comparable (48% vs. 46%). The SG arm had lower rates of treatment discontinuation and dose reduction. Common grade ≥ 3 adverse events (AEs) included neutropenia and diarrhea, indicating manageable tolerability in the first-line treatment.

#### 4.1.3. Correlation of Design with Clinical Profile and Future Directions

The clinical profile of SG stems from its innovative design. Its high DAR and TME-cleavable linker are engineered to maximize intratumoral delivery of SN-38 and its bystander effect, providing the molecular basis for its efficacy [25]. This potent payload delivery also shapes its safety profile, manifesting primarily as neutropenia and diarrhea [30]. Ongoing clinical development is now focused on evaluating SG in combination strategies (e.g., with immune checkpoint inhibitors) and assessing its role in earlier lines of therapy.

Combination strategies have shown promise. The phase II NeoSTAR study (NCT04230109) [33] has established a novel strategy for the neoadjuvant treatment of early-stage TNBC. It demonstrated that after 4 cycles of neoadjuvant SG in combination with pembrolizumab, 34% of patients achieved a pathological complete response (pCR) without receiving any additional chemotherapy. This outcome represents a dual breakthrough: firstly, it marks the first successful extension of SG’s application from the metastatic setting to the early-stage neoadjuvant arena; secondly, the regimen is the first to validate the significant potential of an ADC combined with immunotherapy in early-stage TNBC. The most common treatment-related AEs included nausea (56%), alopecia (52%), and fatigue (46%). In terms of immune combination therapy, The phase III ASCENT-04/KEYNOTE-D19 study (NCT05382286)demonstrated a trend towards improved mPFS with SG plus pembrolizumab versus chemotherapy plus immunotherapy in PD-L1-positive patients (11.2 vs. 7.8 months; HR = 0.65) [34]. Furthermore, the SG combination regimen was associated with lower rates of treatment discontinuation and dose reduction due to adverse events. This phase III study, the first of its kind globally to demonstrate the superiority of an ADC combined with immunotherapy over traditional chemotherapy combined with immunotherapy, was designed to evaluate the efficacy of this combination as first-line treatment in patients with advanced PD-L1-positive (combined positive score [CPS] ≥ 10) TNBC. The MORPHEUS-pan BC study (NCT03424005) [35] demonstrates the Respectable efficacy of SG combined with atezolizumab (a PD-L1 inhibitor) as first-line treatment for PD-L1-positive mTNBC, with an ORR of 76.7% and a significantly prolonged mPFS (12.2 months vs. 5.9 months). Currently, multiple studies are further exploring SG’s combination strategies with different immunotherapies, including the SACI-IO TNBC study (NCT04468061) [36] for PD-L1-negative patients, the ASPRIA study (NCT04434040) [37] for recurrence prevention, and combination regimens for brain metastasis (NCT06238921) [38]. These studies preliminarily confirm the safety and synergistic antitumor activity of SG combined with immunotherapy. In terms of targeted combination therapy, SG followed by the PARPi talazoparib (NCT04039230) [39] achieved an ORR of 30.1% and a mPFS of 7.6 months (vs. 2.3 months). Additionally, the efficacy of SG combined with the CDK4/6 inhibitor trilaciclib (NCT05113966) [40] and the PI3K inhibitor alpelisib (NCT05143229) [41] is still under investigation. In summary, these data collectively indicate that SG, through its unique drug structure and dual mechanism of action, exhibits advantages in both monotherapy and combination therapy for mTNBC, providing an important treatment option for clinical practice.

### 4.2. Datopotamab Deruxtecan (Dato-DXd): Engineering for an Optimized Therapeutic Index

#### 4.2.1. Design Philosophy and Distinctive Features

As a next-generation Trop2-targeted ADC, Datopotamab deruxtecan (Dato-DXd) (Table 3) is designed with a focus on optimizing the therapeutic window. It employs a moderate DAR of 4 and an enzyme-cleavable glycine-glycine-phenylalanine-glycine (GGFG) tetrapeptide linker, conjugated to the Top 1 inhibitor DXd, which exhibits high membrane permeability. This design is engineered to balance plasma stability with potent intracellular cytotoxicity [42], In vitro studies have confirmed that DXd induces caspase-dependent apoptosis in tumor cells by stabilizing Top1cc, leading to double-strand breaks (DSBs) and replication fork collapse [43].

#### 4.2.2. Clinical Development and Emerging Efficacy Data

Clinical activity has been observed across settings. The Phase I basket trial TROPION-PanTumor01 (NCT03401385) evaluated the efficacy and safety of Dato-DXd in multiple advanced solid tumors [44]. In the TNBC cohort (n = 44)—which included patients who had received a median of three prior lines of therapy—the ORR was 31.8%, the disease control rate (DCR) reached 79.5%, mPFS was 4.4 months, and mOS was 14.3 months [45]. In the Ib/II BEGONIA study (NCT03742102), first-line treatment with Dato-DXd combined with durvalumab (DUR) demonstrated efficacy in the mTNBC cohort (n = 62), characterized by an ORR of 79%, an mPFS of 13.8 months, and an mDOR of 15.5 months [46]. In contrast, the Phase III TROPION-Breast01 trial (NCT05104866) investigated its application in patients with hormone receptor-positive (HR+)/human epidermal growth factor receptor 2-negative (HER2-) breast cancer, Dato-DXd significantly prolonged mPFS compared with the investigator’s choice of chemotherapy (6.9 vs. 4.9 months; HR = 0.63), with a lower incidence of grade ≥ 3 treatment-related AEs (21% vs. 45%) [47]. Based on these data, a marketing application for Dato-DXd has been submitted and accepted for review [48] for the treatment of previously treated patients with HR+/HER2-breast cancer. TROPION-Breast02 (NCT05374512) [49] was a Phase III study evaluating Dato-DXd versus investigator’s choice of chemotherapy (ICC: nab-paclitaxel, capecitabine, eribulin, or carboplatin), which included, as first-line treatment for patients with inoperable locally recurrent or mTNBC who were ineligible for immunotherapy. With a median follow-up of 27.5 months, Dato-DXd demonstrated statistically significant and clinically meaningful improvements in both co-primary endpoints of progression-free survival (PFS) and OS, showing superior mPFS (10.8 months vs. 5.6 months) and mOS (23.7 months vs. 18.7 months) compared to ICC. The safety profile of Dato-DXd was manageable, featuring a longer treatment duration alongside a lower discontinuation rate. These results support Dato-DXd as a new standard first-line treatment for this patient population.

#### 4.2.3. Safety Profile and Ongoing Combination Strategie

The distinct engineering of Dato-DXd correlates with its efficacy and a unique toxicity risk of interstitial lung disease (ILD) requiring vigilant monitoring [13]. Furthermore, research on Dato-DXd-based combination therapy in TNBC is progressively advancing. Multiple clinical trials are currently evaluating the efficacy of Dato-DXd ± DUR in different TNBC treatment settings: The Phase III TROPION-Breast05 trial (NCT06103864) targets patients with advanced TNBC (aTNBC) [50], The Phase III TROPION-Breast04 trial (NCT06112379) explores its use in the neoadjuvant/adjuvant setting for TNBC patients [51], The Phase II TROPION-Breast03 trial (NCT05629585) focuses on TNBC patients who did not achieve a pCR after neoadjuvant therapy [52]. Additionally, a preclinical study (P4-03-23) [53] demonstrated that the combination of trastuzumab deruxtecan (T-DXd) with the PARPi olaparib exhibited synergistic antitumor activity in HER2-low/negative TNBC patient-derived xenograft (PDX) models, providing critical evidence for this combination strategy. collectively, these data indicate the clinical activity of Dato-DXd and warrant further investigation in combination therapies for TNBC.

### 4.3. Sacituzumab Tirumotecan (SKB-264): Refinement of the First-Generation Blueprint

#### 4.3.1. Optimized Molecular Design

Sacituzumab tirumotecan (SKB-264) (Table 4) is the first Trop2-targeted ADC developed independently in China. It incorporates refinements upon first-generation Trop2 ADCs. It maintains a high DAR of 7.4 while employing a novel, stable linker (TL033) based on a mesyl-sulfonyl-pyrimidine–CL2A–carbonate architecture, together with a proprietary Top I inhibitor payload (KL610023, also known as T030). This design leverages irreversible antibody conjugation via the mesyl-sulfonyl-pyrimidine group to enhance plasma stability [14]. Under the acidic environment of lysosomes and under the action of proteases, T030 is released. T030 exerts its cytotoxic effect by inhibiting Top I, inducing DNA damage, and ultimately leading to tumor cell apoptosis [54].

#### 4.3.2. Clinical Efficacy Across Treatment Lines

The Phase I/II clinical trial (NCT04152499) demonstrated significant efficacy in 59 patients with TNBC [55]. The confirmed ORR was 46.1% and 62.5% in the 4 mg/kg and 5 mg/kg dose groups, respectively. The primary grade 3 treatment-related AEs were neutropenia (23.7%) and anemia (20.3%). The pivotal Phase III OptiTROP-Breast01 study (NCT0537134, n = 263) confirmed that, compared with chemotherapy, SKB-264 significantly prolonged mPFS (6.7 months vs. 2.5 months; HR = 0.31, 95% CI: 0.22–0.45, *p* < 0.05) and mOS (not reached vs. 9.4 months; HR = 0.53, 95% CI: 0.36–0.78, *p* < 0.05) [56]. Blinded independent central review showed an ORR of 45.4% in the SKB-264 group (compared to 12.0% in the chemotherapy group), which increased to 52.1% in the Trop2-high expression subgroup. Regarding safety, the primary grade 3 treatment-related AEs were neutropenia (32.3%) and anemia (27.7%). Based on these data, SKB-264 has received three Breakthrough Therapy designations, and its New Drug Application (NDA) has been accepted by the NMPA in 2023 [57], The phase II OptiTROP-Breast05 study (NCT05445908) [58] evaluated SKB-264 as first-line treatment for advanced/metastatic TNBC. With a median follow-up of 18.6 months, the primary efficacy outcomes demonstrated an ORR of 70.7% and a DCR of 92.7%. The mPFS was 13.4 months, with a 12-month PFS rate of 64.6%. Notably, substantial efficacy was maintained in the PD-L1 low-expression subgroup. Regarding safety, grade ≥ 3 treatment-related AEs occurred in 63.4% of patients, predominantly hematological toxicities.

#### 4.3.3. Design-Outcome Correlation and Evolving Therapeutic Role

SKB-264’s design retains a high DAR akin to SG for potency but incorporates optimizations for plasma stability and therapeutic index [17,20,56]. This translates into a safety profile with myelosuppression (neutropenia, anemia) similar to SG but potentially a lower incidence of severe diarrhea. Current research is exploring combination therapy strategies for SKB-264. Two Phase III studies targeting first-line treatment of PD-L1-negative (CPS < 10) advanced or metastatic TNBC (a/mTNBC) are underway: a China-based study (NCT06279364): SKB-264 versus investigator’s choice of chemotherapy; and a global study (NCT06841354): SKB-264 ± pembrolizumab versus chemotherapy. A Phase II clinical study is evaluating the efficacy and safety of SKB-264 ± KL-A167 (a PD-L1 inhibitor) in treatment-naïve patients with locally advanced or metastatic TNBC (LA/mTNBC), further expanding its potential for clinical application.

### 4.4. Others

Beyond these agents, a pipeline of early-stage Trop2-targeted ADCs represents diversified engineering strategies aimed at addressing the limitations of first-generation constructs, with a primary focus on enhancing the therapeutic index. These efforts bifurcate into two main directions: linker technology optimization (e.g., ESG401 [NCT04892342], which utilizes stable linker technology [DAR = 8] to conjugate SN-38, aiming to reduce off-target toxicity [59]) and the development of novel payloads to overcome resistance (e.g., DB-1305 [NCT05438329], which conjugates the novel Top I inhibitor P1021 to an anti-Trop2 antibody via a cleavable tetrapeptide linker and has demonstrated significant antitumor activity in breast, colon, and lung cancer models [60]; BL-M02D1 [NCT05339685], which employs the camptothecin derivative Ed-04 [61]). The historical discontinuation of PF-06664178 (NCT02122146) due to dose-limiting toxicities underscores the persistent challenge of balancing efficacy and toxicity [62]. Collectively, these candidates exemplify a “design iteration” centered on linker stability, payload innovation, and DAR optimization, all aimed at expanding future therapeutic options for patients with Trop2-positive cancers.

## 5. Summary and Outlook

### 5.1. Limitations of Current Evidence and Unmet Needs

**Figure 3:**
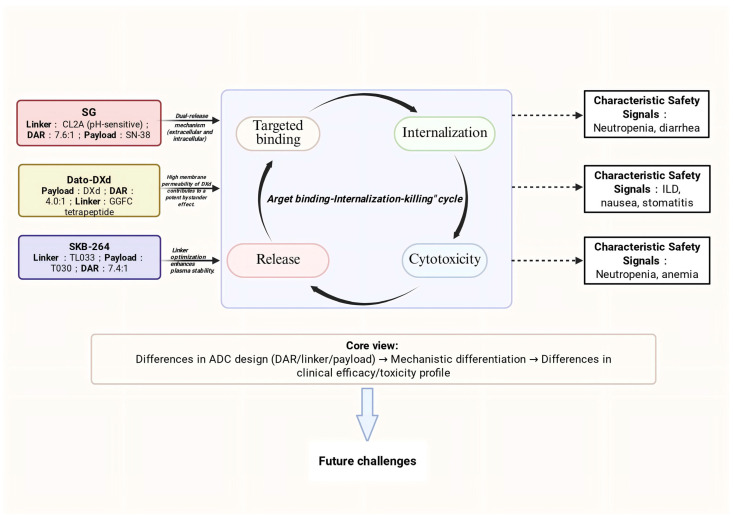
Summary figure: A comparative overview of the design parameters (linker, DAR, payload), mechanisms of action, and characteristic safety signals of three ADCs. This figure highlights how differences in ADCs design drive mechanistic differentiation and lead to distinct clinical efficacy and toxicity profiles, and outlines future challenges in the field. Figure created with BioRender.com.

Trop2-targeted ADCs represent a significant breakthrough in solid tumor therapy, As summarized in Figure 3 (Figure 3), the evolution of this field, from Trop2 biology to the design, mechanisms, and clinical outcomes of various ADCs, demonstrates a clear and interconnected trajectory. Currently, first-generation agents such as SG have achieved clinical success in TNBC, while next-generation candidates (e.g., SKB-264 and Dato-DXd) are enhancing therapeutic windows through technological innovations, including linker optimization and the development of novel payloads. Despite the promising outlook, the field of Trop2 ADCs faces a series of scientific and clinical challenges that constitute critical directions for future research and clinical optimization.

#### 5.1.1. Overcoming Resistance Mechanisms

The emerging issue of drug resistance requires in-depth elucidation. The underlying mechanisms are complex and multifaceted, primarily including: (a) Target-related mechanisms: such as spatial heterogeneity in Trop2 expression or genetic mutations (e.g., T256R), which can prevent effective ADC binding or internalization [63]; (b) Intracellular trafficking and metabolic barriers: including altered lysosomal pH or dysfunction of transporter proteins (e.g., SLC46A3), hindering the effective release of the payload [64,65]; and (c) Enhanced payload efflux: tumor cells may upregulate drug efflux pumps like ABCG2 and ABCB1 (MDR1), actively reducing the intracellular concentration of the cytotoxic payload. These resistance mechanisms may partly explain the observed clinical phenomena where some patients show primary resistance or subsequently develop progressive disease on ADC therapy [66,67]. Furthermore, the sequential use of different ADCs may lead to cross-resistance due to shared targets or payload mechanisms [68], underscoring the need for careful planning of treatment sequences in clinical practice.

#### 5.1.2. Advancing Predictive Biomarkers

Currently, IHC detection of Trop2 protein remains the primary method for patient selection, yet its clinical application faces significant hurdles: a lack of globally unified scoring standards (antibodies, thresholds), results confounded by tumor spatial heterogeneity, and the inability of static IHC scoring to reflect the dynamic changes in Trop2 expression during treatment or the potential impact of post-translational modifications (e.g., glycosylation) on ADC binding efficiency [69,70]. Although studies such as the OptiTROP-Breast01 trial for SKB-264 suggest that high Trop2 expression may be associated with a better ORR [56], static IHC scoring alone remains insufficient for precise individual outcome prediction. Future efforts must focus on developing standardized detection methods, integrating multi-region biopsies or liquid biopsies (e.g., dynamic circulating tumor DNA (ctDNA) monitoring) [71], and exploring evaluation systems that assess the functional status of Trop2—not merely its expression level—for more precise patient stratification.

#### 5.1.3. Managing Differentiated Safety Profiles

As evidenced by the clinical trial data in Section 4, different Trop2 ADCs possess characteristic safety profiles that directly influence clinical management. The phase III ASCENT trial for SG reported grade ≥ 3 neutropenia and diarrhea as predominant toxicities [29]. Clinical data for Dato-DXd warrant special vigilance for the risk of ILD [72,73]. The OptiTROP-Breast01 study for SKB-264 reported significant incidences of grade ≥ 3 neutropenia and anemia [56]. A deep understanding of these differentiated toxicities and the establishment of targeted prevention and management protocols are therefore fundamental. For SG, the most common grade ≥ 3 AEs are neutropenia and diarrhea; however, real-world studies indicate that prophylactic use of granulocyte colony-stimulating factor (G-CSF) and supportive care can significantly reduce their incidence [74,75]. Dato-DXd requires special vigilance for the risk of ILD, in addition to managing common events like stomatitis, ocular toxicity, and infusion-related reactions. SKB-264 management primarily focuses on hematological toxicities (neutropenia, anemia) and stomatitis. A deep understanding of these differentiated toxicities and the establishment of targeted prevention, monitoring, and intervention protocols are fundamental to ensuring patient safety and treatment tolerability.

#### 5.1.4. Prudent Interpretation of Cross-Trial Data: Analysis of Key Trial Design Differences

The rapid clinical translation of Trop2-targeted ADCs presents several interpretive challenges. Current understanding of the efficacy and safety profiles of SG, Dato-DXd, and SKB-264 is derived from several independent pivotal trials. In the absence of direct head-to-head comparisons, cross-trial interpretation must acknowledge fundamental differences in target population, treatment context, and evaluation framework, which limit direct data comparability.

Key design heterogeneities and limitations include:

Differences in Treatment Stage and Patient Baseline: The pivotal trials enrolled patients at distinct stages of disease, aiming to evaluate each drug’s value in different clinical contexts. For instance, the phase III ASCENT trial for SG primarily focused on refractory mTNBC after ≥2 prior lines of therapy [29], while the TROPION-Breast02 trial for Dato-DXd and the OptiTROP-Breast05 study for SKB-264 evaluated efficacy in the first-line treatment [49,58]. The expected survival and baseline treatment response inherently differ among populations at different treatment lines.

Uncertainty Regarding Optimal Treatment Strategy and Sequencing: As these trials were designed independently and focused on specific lines of therapy, the optimal sequencing of these ADC agents both among themselves and in relation to other standard therapies (e.g., immune checkpoint inhibitors, PARPi) remains a critical and unresolved clinical question [68].

Varying Criteria for Defining Study Populations: Trials employed different biomarkers (e.g., PD-L1) for screening or stratification, meaning they address fundamentally different clinical questions. For example, studies limiting enrollment to PD-L1-positive patients (e.g., The phase III ASCENT-04/KEYNOTE-D19 study) have fundamentally different applicability of their results compared to those without such restrictions [34].

Divergent Contexts for Efficacy Evaluation: The choice of primary endpoint (e.g., PFS versus PFS/OS co-primary) reflects different research objectives. Moreover, the specific composition of the control arm regimen and whether cross-over was permitted significantly influence the interpretation of the magnitude and attribution of survival benefit [49].

Diversity and Methodological Limitations in Study Design: For instance, some trials are underpowered due to limited sample sizes [44,55], which compromises the precision of treatment effect estimates and the detection of rare AEs. Observational studies are prone to selection bias and baseline imbalances, potentially leading to confounding by indication that obscures true efficacy assessments [76]. Furthermore, issues with follow-up adherence contribute to attrition bias, undermining the completeness and reliability of outcome data. Finally, concerns regarding limited external validity (EV) remain, further compounded by the relatively short follow-up in trials of newer agents and the paucity of robust real-world evidence, which together constrain the assessment of long-term outcomes and generalizability [77], as findings derived from controlled trial settings may not be fully generalizable to the broader, more heterogeneous patient populations seen in routine clinical practice.

### 5.2. Future Therapeutic Strategies and Directions

Confronting tumor heterogeneity and resistance, combination therapy is an inevitable trend. The synergistic effects of SG combined with immune checkpoint inhibitors in both advanced and neoadjuvant settings have been preliminarily validated. Combination strategies of Dato-DXd or SKB-264 with immunotherapy or targeted agents (e.g., PARPi) are also under active exploration. Regarding treatment strategy, network meta-analyses suggest that SG may offer superior monotherapy survival benefits in pretreated aTNBC [78]. Clinical decision-making should comprehensively consider tumor molecular characteristics (e.g., Trop2 expression level and distribution, PD-L1 status), prior treatment history (especially previous chemotherapy and ADC exposure), patient comorbidities, and tolerance to different toxicity profiles to individualize the choice of agent and its timing of application (first-line, second-line, or sequential).

Looking ahead, key directions for advancing the ADC therapeutic landscape in TNBC include:

(1) Structure-based rational drug design to overcome the current limited understanding of the full-length Trop2 protein activation mechanism and to facilitate the development of more selective next-generation Trop2 ADCs.
(2) Exploring target diversity beyond Trop2.

Given the high heterogeneity of TNBC, ADCs targeting other tumor-associated antigens are showing promise in early-stage clinical research. Among these, HER2 represents a significant target, with low expression present in approximately 50% of TNBC cases. The novel HER2-targeted ADCs, T-DXd, has demonstrated remarkable progress [79]. T-DXd is composed of trastuzumab linked to the Top I inhibitor DXd via a cleavable linker, with a DAR of approximately 8. The pivotal Phase III DESTINY-BREAST04 trial showed that in previously treated patients with HER2-low advanced breast cancer (including a TNBC subgroup), T-DXd significantly improved PFS and OS compared to chemotherapy, with an ORR exceeding 50%, although its primary AEs were hematologic toxicities [80]. The Phase II DAISY study further confirmed the antitumor activity of T-DXd in patients with HER2-low and HER2-zero expression (including TNBC) [81]. Beyond HER2, explorations targeting other antigens are underway. For instance, patritumab deruxtecan (HER3-DXd) targeting HER3 demonstrated an ORR of 22.6% in a Phase I/II study involving TNBC [82]; agents such as HS-20089 targeting B7-H4 and ladiratuzumab vedotin (LV) targeting LIV-1 have also shown preliminary antitumor activity [83,84]. These explorations may provide new potential options for patients with low Trop2 expression or those unsuitable for Trop2-targeted therapy.

(3) Developing novel ADC technology platforms, such as bispecific ADCs and prodrug-based ADCs, to overcome resistance and expand the therapeutic window [85].
(4) Deepening translational research and advancing intelligent clinical trial design: Utilizing patient-derived models and real-world data to elucidate resistance mechanisms and validate novel biomarkers; designing dynamic, biomarker-guided treatment strategies and optimal sequential or combination regimens involving different ADCs (including those with diverse targets).

Through continuous platform optimization, target expansion, and mechanistic exploration, ADC therapies are poised to further solidify their cornerstone role in the precision medicine of TNBC and solid tumors at large, ultimately delivering superior survival outcomes for patients.

## 6. Conclusions

Trop2-targeted ADCs have emerged as a significant breakthrough in the treatment of TNBC, demonstrating substantial clinical efficacy, particularly in advanced and treatment-resistant patients. Moving forward, their clinical value is expected to be further enhanced through structural optimization, combination strategies, and the development of predictive biomarkers, thereby advancing the field of precision therapy for TNBC.

## Figures and Tables

**Table 1:** Comparison of three drugs.

Feature	SG	Dato-DXd	SKB-264
Trop-2 mAb	Humanized IgG1	Humanized IgG1	Humanized IgG1
Linker	CL2A (pH-sensitive)	GGFG tetrapeptide (enzyme-sensitive)	TL033 (pH-sensitive, optimized from CL2A)
Payload	SN-38 (active metabolite of irinotecan)	DXd (derivative of exatecan)	T030 (derivative of belotecan)
DAR	7.6:1	4.0:1	7.4:1
Primary Release Mechanism	Lysosomal acidic environment + tumor extracellular cleavage	Intratumoral cellular lysosomal protease cleavage	Lysosomal acidic environment
Pharmacological Focus	Potent bystander effect, dual release mechanism	High plasma stability, precise intracellular release, highly active payload	Optimized plasma stability, high DAR, potent payload
Characteristic Safety Signals	Neutropenia, diarrhea	ILD, nausea, stomatitis	Neutropenia, anemia

mAb: monoclonal antibody; DAR: drug-to-antibody ratio; ILD: interstitial lung disease; GGFG: glycine-glycine-phenylalanine-glycine.

**Table 2:** Major Clinical Studies on SG.

ID	Early or Advanced Stages	Research Staging	Endpoint	Key Findings	Treatment	Enrolled Patients
Monotherapy						
NCT01631552	advanced stages	The Phase I/II IMMU-132-01 study	ORR	ORR: 33.3%, mPFS: 5.5 months, mOS: 13 months	SG	mTNBC patients
NCT02574455	advanced stages	The Phase III ASCENT study	PFS	mPFS: 5.6 months vs. 1.7 months; mOS: 12.1 months vs. 6.7 months;	SG vs. TPC ^a^	Patients with mTNBC Who received at least two prior treatments
NCT05382299	advanced stages	The Phase III ASCENT-03 study	PFS	mPFS: 9.7 vs. 6.9 months; mDOR: 12.2 vs. 7.2 months	SG vs. TPC ^b^	Patients with previously untreated advanced TNBC(aTNBC) who are unable to receive PD-(L)1 Inhibitors ^c^
Combination Therapy						
NCT04230109	Early stages	The phase II NeoSTAR study	pCR	pCR: 34%	SG + Pembrolizumab	Early stage TNBC patients without prior treatment
NCT05382286	advanced stages	The ASCENT-04 study	PFS	mPFS: 11.2 vs. 7.8 months	SG + pembrolizumab vs. TPC + pembrolizumab	Patients with untreated LA/mTNBC ^d^ who have PD-L1-positive tumors (CPS ≥ 10)
NCT03424005	advanced stages	The MORPHEUS-pan BC study	ORR	ORR: 76.7% vs. 66.7%; mPFS: 12.2 vs. 5.9 months	Atezo + SG vs. Atezo + nabP	Patients with untreated, PD-L1-positive, unresectable LA/mTNBC.

mPFS: median Progression-free survival; ORR: Objective Response Rate; mOS: median Overall Survival; mDOR: median duration of response; SG: Sacituzumab govitecan; Atezo: atezolizumab; ^a^ TPC: physician’s choice chemotherapy (eribulin, vinorelbine, gemcitabine, or capecitabine); ^b^ TPC: physician’s choice chemotherapy(Paclitaxel, nab-Paclitaxel or Gemcitabine + Carboplatin); ^c^ Patients are unable to receive PD-(L)1 Inhibitors ^c^: PD-L1-negative tumors(combined positive score [CPS] < 10), PD-L1-positive tumors(CPS10) and previously treated with a PD-(L)1 inhibitor in curative setting Ineligible for a PD-(L)1 inhibitor due to a comorbidity; ^d^ LA/mTNBC: locally advanced or metastatic TNBC.

**Table 3:** Major Clinical Studies on Dato-DXd.

ID	Early or Advanced Stages	Research Staging	Endpoint	Key Findings	Treatment	Enrolled Patients
Monotherapy						
NCT03401385	advanced stages	The Phase I basket trial TROPION-PanTumor01 study	Safety and Tolerability	ORR: 31.8%, mPFS: 4.4 months, mOS: 14.3 months	Dato-DXd	Advanced/unresectable or metastatic HR+/HER2-BCor HR-/HER2-(TNBC) Relapsed or progressed after local standard treatments
NCT05104866	advanced stages	The Phase III TROPION-Breast01 study	PFS	mPFS: 6.9 vs. 4.9 months	Dato-DXd vs. ICC	Patients with unresectable/metastatic HR+/HER2-breast cancer, post-progression on or ineligible for endocrine therapy, and with 1–2 prior lines of metastatic chemotherapy.
NCT05374512	advanced stages	The TROPION-Breast02 Phase III study	PFS and OS	mPFS: 10.8 vs. 5.6 months and mOS: 23.7 vs. 18.7 months	Dato-DXd vs. ICC	Patients with untreated TNBC that is either unresectable locoregionally recurrent or metastatic, and who are ineligible for immunotherapy.
Combination Therapy						
NCT03742102	advanced stages	The Ib/II BEGONIA study	Safety and Tolerability	ORR: 79% mPFS: 13.8 months	Dato-DXd + DUR	First-line treatment for patients with unresectable a/mTNBC ^a^.

Dato-DXd: Datopotamab deruxtecan; ICC: investigator’s choice of chemotherapy (nab-paclitaxel, capecitabine, eribulin, or carboplatin); DUR: durvalumab; mPFS: median Progression-free survival; ORR: Objective Response Rate; mOS: median Overall Survival; ^a^ a/mTNBC: advanced or metastatic TNBC.

**Table 4:** Major Clinical Studies on SKB-264.

ID	Early or Advanced Stages	Research Staging	Endpoint	Key Findings	Treatment	Enrolled Patients
Monotherapy						
NCT04152499	advanced stages	The Phase I/II clinical study	OS	ORR (4 mg/kg vs. 5 mg/kg): 46.1% vs. 62.5%	SKB-264	Advanced HER2-negative breast cancer
NCT0537134	advanced stages	The pivotal Phase III OptiTROP-Breast01 study	PFS	mPFS: 6.7 months vs. 2.5 months	SKB-264 vs. TPC	Patients with LA/mTNBC ^a^ previously treated with ≥2 chemotherapy regimens
NCT05445908	advanced stages	The Phase II OptiTROP-Breast05 study	ORR and DCR	ORR: 70.7% DCR: 92.7%. mDoR: 12.2 months mPFS: 13.4 months	SKB-26	Patients with a/mTNBC who have not received prior treatment for advanced disease (regardless of CPS or Trop2 expression status)

SKB-264: Sacituzumab tirumotecan; TPC: physician’s choice chemotherapy (eribulin, vinorelbine, gemcitabine, or capecitabine); mPFS: median Progression-free survival; ORR: Objective Response Rate; mDOR: median duration of response; DCR: disease control rate; ^a^ LA/mTNBC: locally advanced or metastatic TNBC.

## Data Availability

No new data were created or analyzed in this study. Data sharing is not applicable to this article.
